# Dr. William J. Whitford

**Published:** 1912-06

**Authors:** 


Dr. William J. Whitford of Schenevus, N. Y., died April 19, aged 55. He was a graduate of Vermont, 1889.

